# Saturn's dayside ultraviolet auroras: Evidence for morphological dependence on the direction of the upstream interplanetary magnetic field

**DOI:** 10.1002/2013JA019598

**Published:** 2014-03-26

**Authors:** C J Meredith, I I Alexeev, S V Badman, E S Belenkaya, S W H Cowley, M K Dougherty, V V Kalegaev, G R Lewis, J D Nichols

**Affiliations:** 1 Department of Physics and Astronomy, University of LeicesterLeicester, UK; 2 Lomonosov Moscow State University, Skobeltsyn Institute of Nuclear PhysicsMoscow, Russian Federation; 3 Department of Physics, University of LancasterLancaster, UK; 4 Blackett Laboratory, Imperial College LondonLondon, UK; 5 Mullard Space Science Laboratory, University College LondonDorking, UK

**Keywords:** Auroral phenomena, Hubble Space Telescope, Saturn, Solar wind dependence

## Abstract

We examine a unique data set from seven Hubble Space Telescope (HST) “visits” that imaged Saturn's northern dayside ultraviolet emissions exhibiting usual circumpolar “auroral oval” morphologies, during which Cassini measured the interplanetary magnetic field (IMF) upstream of Saturn's bow shock over intervals of several hours. The auroras generally consist of a dawn arc extending toward noon centered near ∼15° colatitude, together with intermittent patchy forms at ∼10° colatitude and poleward thereof, located between noon and dusk. The dawn arc is a persistent feature, but exhibits variations in position, width, and intensity, which have no clear relationship with the concurrent IMF. However, the patchy postnoon auroras are found to relate to the (suitably lagged and averaged) IMF *B_z_*, being present during all four visits with positive *B_z_* and absent during all three visits with negative *B_z_*. The most continuous such forms occur in the case of strongest positive *B_z_*. These results suggest that the postnoon forms are associated with reconnection and open flux production at Saturn's magnetopause, related to the similarly interpreted bifurcated auroral arc structures previously observed in this local time sector in Cassini Ultraviolet Imaging Spectrograph data, whose details remain unresolved in these HST images. One of the intervals with negative IMF *B_z_* however exhibits a prenoon patch of very high latitude emission extending poleward of the dawn arc to the magnetic/spin pole, suggestive of the occurrence of lobe reconnection. Overall, these data provide evidence of significant IMF dependence in the morphology of Saturn's dayside auroras.

**Key Points:**

- We examine seven cases of joint HST Saturn auroral images and Cassini IMF data
- The persistent but variable dawn arc shows no obvious IMF dependence
- Patchy postnoon auroras are present for northward IMF but not for southward IMF

## 1. Introduction

It has long been supposed that the dynamics of the magnetospheres of the gas giant planets are dominated by the rotational flows imposed by ion-neutral collisions in the planetary ionosphere [e.g., *Brice and Ioannidis*, 1970; *Kennel and Coroniti*, 1975]. Nevertheless, this expectation does not exclude the possibility that the interaction with the solar wind at the magnetopause boundary produces significant effects involving the outer magnetosphere and magnetospheric tail [e.g., *Cowley et al*., 2003, 2004a]. For the case of Saturn, the topic of the present paper, the estimates of the dayside reconnection rate based on empirical formulae derived from terrestrial experience, indicates typical voltages as low as ∼25 kV during low-field solar wind rarefaction regions, rising to ∼150 kV during high-field compression regions [*Jackman et al*., 2004]. Such rates are thus near negligible in the former case, but competitive with ring current region and outer magnetosphere rotational voltages of a few hundred kilovolts each in the latter (the overall rotational voltage at Saturn however, including the inner magnetosphere, is ∼12 MV) [*Badman and Cowley*, 2007]. These dayside reconnection rates nevertheless imply that the time scale governing the solar wind interaction via open flux production is long. To replenish the tail with a typical ∼30 GWb of open flux would take ∼14 days during solar wind rarefaction regions, reducing to ∼2.5 days during compression regions, such time scales being compatible with the rates at which Saturn's auroral oval is observed to increase in size [*Badman et al*., 2005]. Rapid reductions in open flux at Saturn are then found to be excited by strong impulsive compressions of the magnetosphere by the solar wind, resulting in major poleward expansions of the auroras, which are centered in the dawn sector due to the effect of rapid planetary rotation [*Prangé et al.*, 2004; *Clarke et al*., 2005; *Cowley et al*., 2005; *Nichols et al*., 2013]. Similar compression-induced closure events are also sometimes observed at Earth, then centered nearer to midnight [e.g., *Boudouridis et al*., 2004].

Recent work has emphasized however that beyond the simple scaling assumptions employed by *Jackman et al*. [2004] for Saturn and *Nichols et al*. [2006] for Jupiter, dayside reconnection may differ somewhat between the Earth and the gas giants due to the differing physical conditions occurring in the magnetopause regions on both sides of the boundary. In particular, consideration of the effect of flow shear across the boundary related to the interior planetary rotational flow suggests that reconnection may be favored in the dusk sector, where the flow shear is reduced rather than in the dawn sector where it is maximized [*Desroche et al*., 2013], while effects associated with the plasma pressure gradient across the boundary may often restrict the process to loci, where the magnetospheric and magnetosheath fields are quite closely antiparallel [*Masters et al*., 2012]. In addition, *Lai et al*. [2012] have confirmed using Cassini data that Earth-like, few minute, flux transfer events (FTEs) are absent at Saturn; these representing the signature of transient magnetopause reconnection events that make an important contribution to the overall open flux transport into the tail at Earth [e.g., *Milan et al*., 2000a]. Nevertheless, in addition to the circumstantial evidence for the occurrence of dayside reconnection noted above concerning variations in the size of Saturn's auroral oval, the signatures of reconnection-related plasma heating and magnetic flux connection across the boundary have been reported in Cassini data by *McAndrews et al*. [2008] and *Badman et al*. [2013], while the presence of magnetosheath plasma in the dayside cusp exhibiting plasma injection signatures has been reported by *Radioti et al*. [2013].

In addition to these indicators, *Radioti et al*. [2011] have presented evidence in Cassini Ultraviolet Imaging Spectrograph (UVIS) data for reconnection-related auroral features in Saturn's dayside ultraviolet (UV) auroras, taking the form of sequential extended bifurcations in the auroral oval in the noon to dusk sector. We noted above that the dusk sector is favored for reconnection at Saturn due to the reduced flow shear across the boundary, between subcorotating magnetospheric plasma on the inside and antisunward flowing magnetosheath plasma on the outside. The auroral bifurcations are found to recur on ∼1–2 h time scales and endure as discrete features for comparable or longer intervals, so that more than one such feature is often present simultaneously, moving slowly poleward and eastward at ∼15% of rigid corotation [*Radioti et al*., 2013]. In the events studied by *Radioti et al*. [2011], the largest “arcs” were found to contain ∼2 GWb of magnetic flux, corresponding to ∼10% of the preexisting flux lying poleward of the auroral oval, with the oval boundary at other local times expanding equatorward accordingly as the arcs moved poleward. An overall increase in open flux of ∼6 GWb over a ∼4 h interval (see their Figure 4c) corresponds to a substantial averaged dayside reconnection rate of ∼400 kV in this case, rather larger than the typical values estimated by *Jackman et al*. [2004] noted above. Similar auroral structures have also been reported by *Badman et al*. [2012] in infrared emissions observed by the Cassini visual and infrared mapping spectrometer (VIMS), but in this case centered nearer to noon.

Morphologically, these auroral features have strong similarities with the dayside auroras associated with FTEs at Earth [*Milan et al*., 2000a], but in the latter case having recurrence times of ∼5–10 min and lasting for ∼10–20 min, so that more than one event is also typically present at any given time. It seems likely that the difference in time scales between Earth and Saturn lies in the different spatial scales of these systems combined with similar plasma and field line propagation speeds. In particular, a time scale relevant to the lifetime of dayside auroral features is the time required for open flux tubes (specifically their magnetopause intersection point) to propagate from the dayside reconnection site into the lobe of the tail. Newly opened flux tubes generate ionospheric flow, field-aligned current, and precipitation that result in dayside auroras, but these die away as the flux tubes are transported downtail, are assimilated into the tail lobe, and become aurorally dark [e.g., *Cowley and Lockwood*, 1992; *Milan et al*., 2000a]. Taking for simplicity, the downtail path length on the nightside to be comparable to that on the dayside, appropriate to the size of the system, the overall flux tube propagation path will typically be ∼35 Earth radii (*R_E_*) at Earth and ∼65 Saturn radii (*R_S_*) at Saturn, for typical subsolar magnetopause radial distances of ∼11 *R_E_* and ∼21 *R_S_*, respectively. With similar flux tube propagation speeds comparable to a typical solar wind speed of ∼450 km s^−1^, the relevant time scales are thus ∼10 min at Earth and ∼2.5 h at Saturn. If the *Radioti et al*.'s [2011, 2013] events correspond to FTEs at Saturn, as seems likely, it is then unsurprising that the study of magnetopause phenomena by *Lai et al*. [2012], looking for features occurring on terrestrial time scales, failed to detect any. In support of this suggestion, *Radioti et al*. [2013] have shown that when Cassini was located inside the magnetosphere and near conjugate to one such arc, it was immersed in magnetosheath-like cusp plasma near to its poleward exit into the polar “plasma void” region beyond. Similarly, in a related auroral case study when Cassini was located nearer to the magnetopause, *Badman et al*. [2013] have shown that the simultaneous near-noon magnetosheath field was directed northward, favoring lower latitude reconnection and open flux production at Saturn, with signatures present of escaping magnetospheric electrons. We note in this regard that Saturn's magnetic dipole is directed parallel to the planet's spin axis, opposite to the case of the Earth, such that open flux production is favored for northward directed interplanetary magnetic field (IMF) at Saturn rather than for southward IMF at Earth.

In this case, we should expect the morphology of Saturn's dayside auroras to depend on the sense of the upstream IMF, in a similar manner to that previously discussed by *Bunce et al*. [2005] and *Gérard et al*. [2005]. Specifically, for northward IMF-favoring lower latitude reconnection, we should expect auroral dynamics to occur on newly opened field lines as discussed above, consisting principally of multiple oval bifurcations in the noon to dusk sector produced by pulsed reconnection if the results of *Radioti et al*. [2011, 2013] and *Badman et al*. [2013] represent a guide. For southward IMF, we would then expect such features to disappear, while auroral patches at high latitudes, poleward of the dayside oval, may then occur in association with reconnection between the IMF and the open tail lobe field [e.g., *Milan et al*., 2000b], i.e., “lobe reconnection,” possibly corresponding to the emissions reported by *Badman et al*. [2012].

Opportunities to test these expectations are very limited, however due to a lack of auroral images combined with concurrent suitably lagged upstream IMF values. Examination has first shown that available Cassini UVIS and VIMS images provide little detailed information under these circumstances, due to limited spatial resolution during intervals when Cassini was located beyond the bow shock, combined with near-equatorial viewing geometries. A significant catalogue of UV images is however available from Hubble Space Telescope (HST) observations. *Belenkaya et al*. [2010] studied the southern hemisphere UV auroras observed during the 2008 HST Saturn campaign, when Cassini was located near apoapsis in the solar wind. However, this interval appears to have been somewhat disturbed, with bright auroras extending to high latitudes in the dawn sector as mentioned above, while here we wish to focus attention on more usual auroral oval morphologies under quieter conditions. No simultaneous IMF and image data were obtained during the 2009 Saturn equinox HST campaign [e.g., *Nichols et al*., 2009] and none either in the most recent 2013 HST campaign [e.g., *J. D. Nichols et al*., 2013]. However, during the postequinox 2011 and 2012 HST Saturn campaigns, thus observing northern auroras, we have found seven imaging intervals exhibiting usual oval emissions when Cassini simultaneously lay in the solar wind measuring the upstream IMF over intervals of at least several hours. An additional imaging interval in the 2011 campaign, which exhibited dawn emissions extending toward the pole indicative of magnetospheric compression effects, was excluded from the study. Of the seven cases exhibiting usual ovals, four have northward concurrent IMF and three southward. Here we examine these images to determine to what extent the above morphological expectations are fulfilled. In section 4, we also compare our findings with the results of a parallel study of these images by E. S. Belenkaya et al. (Magnetospheric magnetic field modelling for the 2011 and 2012 HST Saturn aurora campaigns - implications for auroral source regions, submitted to *Annales Geophysicae*, 2014), where the bright auroral features have been mapped along magnetic field lines using the “paraboloid” model of Saturn's magnetospheric magnetic field, which employs the concurrent Cassini IMF values as input.

## 2. Cassini IMF Data

### 2.1 Propagation Delay Between Cassini IMF Measurements and Dayside Auroral Response

In this section, we describe the Cassini IMF measurements used in this study and begin by discussing the propagation delay that associates a HST auroral image obtained at a particular time (corrected for light travel between Saturn and Earth) with a particular interval of IMF data measured by Cassini. Evidently, the IMF which is influencing specifically the dayside auroral morphology at a particular time, is measured at an earlier time due to the frozen-in propagation of the IMF from the spacecraft to the dayside magnetopause, together with the one-way Alfvénic communication time along outer magnetospheric field lines from potential reconnection sites to the ionosphere. The frozen-in propagation time consists of the time from the spacecraft to the bow shock in the upstream solar wind, plus the transit time through the magnetosheath. We now estimate these times using a similar procedure to that employed by *Nichols et al*. [2007] in a study of HST images and concurrent solar wind data during the Cassini Jupiter flyby in 2000/2001.

For the solar wind segment, we reasonably assume that the phase fronts of IMF variations in the solar wind are near perpendicular to the planet-Sun line, the average field spiral angle at these distances also being within a few degrees of perpendicularity. This time is therefore given by *τ*_SW_ =  (*X*_Cass_  −  *R*_BS_) / *V*_SW_, where *X*_Cass_ is the *X* position of the spacecraft in Kronocentric Solar Magnetospheric (KSM) coordinates, *R*_BS_ is the subsolar radial distance of Saturn's bow shock, and *V*_SW_ is the antisunward speed of the solar wind. (In KSM coordinates, *X* points from the center of the planet toward the Sun, the *X–Z* plane contains the planet's magnetic/spin axis, and *Y* completes the orthogonal right-handed set pointing toward dusk. We note that for Saturn, the magnetic and spin axes are coaligned to within measurement uncertainties of ∼0.1° [*Burton et al*., 2010].) Unfortunately, solar wind plasma parameters cannot routinely be determined from Cassini ion data due to instrument pointing limitations [*Young et al*., 2004]. Noting however that the auroral images examined do not include “disturbed” morphologies in the sense discussed in section 1, here we make estimates based on typical values. Specifically, we use a typical solar wind speed and dynamic pressure of ∼450 km s^−1^ and ∼0.03 nPa [e.g., *Arridge et al*., 2006], respectively, to determine typical positions of the bow shock and magnetopause (these values implying a typical solar wind number density of ∼0.08 cm^−3^ at Saturn). The typical subsolar radial distance of the bow shock, given in Saturn radii by *Masters et al*. [2008] as 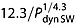, where *P*_dyn SW_ is the dynamic pressure of the solar wind, is then ∼27.8 *R_s_* (Saturn's 1 bar equatorial radius *R_s_* is 60,268 km), such that the solar wind delay in hours is given by *τ*_SW_ ≈ 0.037 × (*X*_*Cass*_(*R*_*S*_) − 27.8). This expression can take negative values if Cassini is located in the solar wind off the planet-Sun line at a smaller *X* than the subsolar shock, as was the case for both the campaign intervals examined here.

To determine the magnetosheath propagation time, we also need the position of the subsolar magnetopause given in Saturn radii by *Kanani et al.* [2010] as 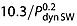, thus equal to ∼20.8 *R_s_* for the above dynamic pressure. The propagation time in the magnetosheath is taken by the formulation of *Khan and Cowley* [1999], in which the speed of the magnetosheath plasma is taken to decrease linearly from a shocked speed of one quarter of the solar wind speed just downstream of the subsolar shock (appropriate to the high Mach number (*M*∼10) solar wind at Saturn) [see, e.g., [*Kivelson and Russell*, 1995], section 5.2.4) to a speed *V*_MP_ at the magnetopause, the latter representing the inflow speed normal to the magnetopause at near-subsolar reconnection sites. The typical value of *V*_MP_ is unknown in detail, but may reasonably be taken to be a few tens of km s^−1^, consistent with the reconnection rates of several tens of kilovolts [e.g., *Jackman et al*., 2004; *Badman et al*., 2005] imposed along a magnetopause reconnection line of length, say ∼10 *R_S_*. Here for definiteness, we have taken a value of 30 km s^−1^, with a resulting propagation delay of *τ*_*Sh*_  ≈  1.9 h. However, the result is not very sensitive to this choice unless *V*_MP_ is taken to become very small.

Finally, rough estimates of the one-way Alfvénic propagation time from the reconnection sites to the ionosphere at Saturn have been made by *Meredith et al*. [2013]. These estimates recognize that such sites may generally be located off equator due to the requirement of near-antiparallel magnetic fields across the magnetopause, the sense of the displacement then depending on the sense of the IMF sector, “toward” or “away.” The estimated propagation times will then be less on the “short” field branch, ∼15 min, than on the “long” field branch, ∼1 h. Here for simplicity, we have taken a fixed compromise propagation time of *τ*_Alf_  ≈  0.5 h. Adding this to the fixed magnetosheath propagation time of ∼1.9 h and the solar wind propagation time to the shock, the overall time delay (lag) from Cassini to the subsolar ionosphere is thus taken to be given by 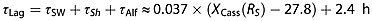
1

Values are ∼1.7 h and ∼2.2 h for the 2011 and 2012 campaign images, respectively, with estimated uncertainties of ∼ ±0.4 h, obtained by considering the usual range of variation of solar wind parameters at Saturn [e.g., *Arridge et al*., 2006], combined in quadrature with the comparable uncertainty in the Alfvénic propagation delay to the ionosphere.

The dayside auroral morphology is not just determined by the instantaneous value of the IMF at this earlier time, however but by a history of the IMF over a preceding interval comparable to the lifetime of auroral features produced by magnetopause dynamics. Following the discussion of the time scales of postnoon arc bifurcations and Saturn FTEs in section 1, this time scale should clearly be taken to be a few hours, and for definiteness is taken here to be 2.5 h. As indicated in section 1, this corresponds to the typical propagation time of open flux tubes over the magnetopause from reconnection sites on the dayside to comparable distances downtail from the planet on the nightside, such that it accommodates additional possible delays resulting from displacements of the reconnection sites away from the subsolar region toward dusk and also largely subsumes the ∼ ±0.4 h uncertainties in the lag time estimates. We thus employ the Cassini IMF data for each HST image centered at Saturn time *t* over a 2.5 h interval from *t*  −  (*τ*_Lag_  +  2.5 h) to *t*  −  *τ*_Lag_ (during which *X*_Cass_ hardly changes), noting that the results are insensitive to the exact interval chosen over a reasonable range. The IMF value averaged over this interval is then taken to be representative of the “concurrent” IMF for a given image. We note however that such intervals relate specifically to the expected time scale for dayside auroral morphology response. As discussed in section 1, the time scale for other responses, such as the large-scale size of the auroral oval determined by the amount of open flux present, will usually be significantly longer, days not hours in this case [*Jackman et al*., 2004; *Badman et al*., 2005], although intervals of somewhat more rapid change may also occur [*Radioti et al*., 2011].

### 2.2. Cassini IMF Data During the 2011 and 2012 HST Saturn Campaigns

**Figure 1:**
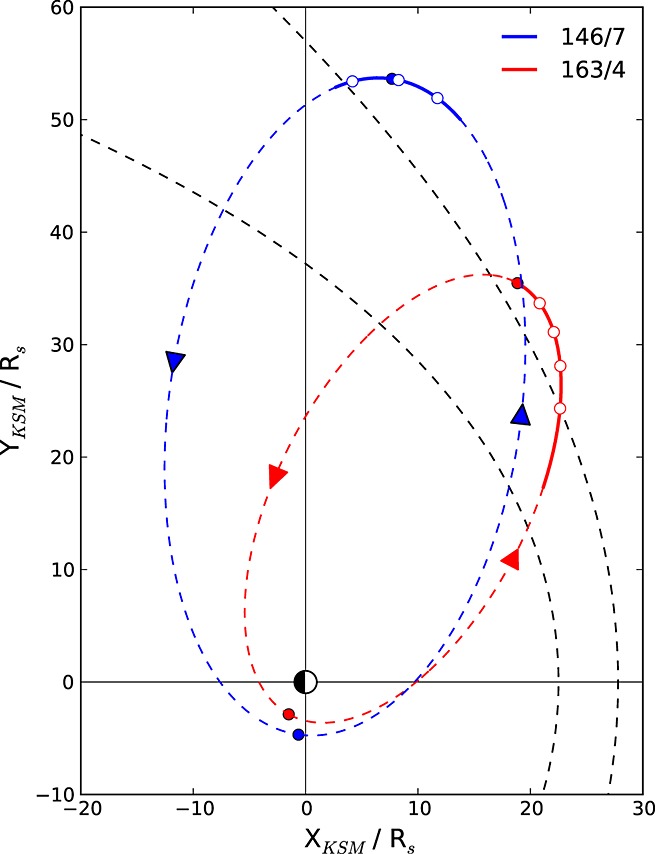
Plot showing the Cassini trajectory projected onto the KSM *X–Y* plane (units of *R_S_*) on two orbits during which the 2011 and 2012 HST Saturn UV auroral campaigns took place. The 2011 campaign took place during the orbit shown by the blue trajectory (Revs 146 outbound and 147 inbound), while the 2012 campaign took place during the orbit shown by the red trajectory (Revs 163 outbound and 164 inbound). Filled circles show apoapsis and periapsis positions on these orbits, while the arrows indicate the direction of spacecraft travel. The solid segments of these trajectories indicate 6 day intervals near apoapsis for which Cassini magnetic field and electron data are shown in Figures 2 and 3. The dashed black lines indicate typical magnetopause (inner) and bow shock (outer) locations for a solar wind dynamic pressure of 0.03 nPa according to the empirical models of *Kanani et al.* [2010] and *Masters et al*. [2008], respectively, showing that the spacecraft is expected to have been located in the magnetosheath or solar wind near the bow shock during these intervals. The unfilled circles indicate the positions of the spacecraft at the center times of seven ∼44 min HST imaging sequences examined in this study, for which the spacecraft was located in the solar wind continuously over several hours.

**Figure 2:**
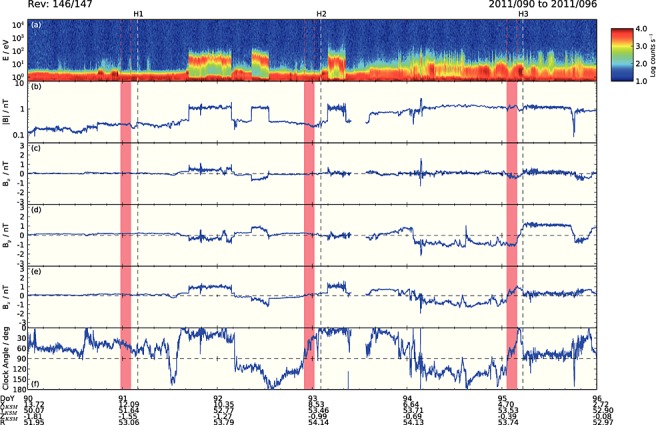
Plot showing Cassini data for the 6 day interval during the 2011 HST Saturn auroral campaign shown by the blue solid line segment of the trajectory in Figure 1, spanning days 90–95, inclusive. (a) A color-coded count rate spectrogram of plasma electrons from 0.6 eV–28.75 keV, (b) the magnetic field strength shown on a log scale (nT), (c–e) the KSM *B_x_*, *B_y_*, and *B_z_* magnetic field components (nT), and (f) the clock angle of the magnetic field about the *X* axis (the planet-Sun direction) given by tan^−1^(*B_y_*/*B_z_*) defined between 0° and 180°. Spacecraft position data are given at the bottom of the figure at the start of each day, specifically its position in Cartesian KSM coordinates, together with the total radial distance from the planet (*R_S_*). Vertical dashed lines indicate the light travel time corrected center times of ∼44 min HST observing visits during the campaign, with visit identifiers indicated at the top of the figure. Red stripes indicate the lagged 2.5 h intervals used to determine the mean concurrent IMF vectors associated with the imaging intervals, as discussed in section 2.1.

**Figure 3:**
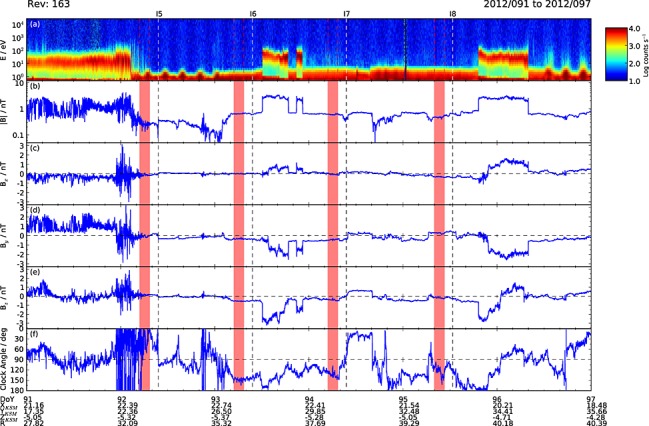
Plot showing Cassini data for the 6 day interval during the 2012 HST Saturn auroral campaign shown by the red solid line segment of the trajectory in Figure 1, corresponding to DOY 91–96, inclusively. The format is the same as Figure 2.

The data employed in this study were obtained during the 2011 and 2012 HST Saturn campaigns, undertaken during intervals when Saturn was in near opposition at Earth. During these intervals, the Cassini trajectory was near equatorial with apoapsis in the predusk sector as shown in Figure 1, where the relevant orbits have been projected into the *X–Y* KSM plane (units of *R_S_*). Here the blue and red dashed lines show the orbits relevant to the 2011 and 2012 campaigns, respectively, with apoapses and periapses indicated by filled circles. Given that Cassini orbit revolution or “Rev” numbers are defined from apoapsis to apoapsis, the orbits correspond to Revs 146 (outbound) and 147 (inbound) for the 2011 campaign and 163 (outbound) and 164 (inbound) for the 2012 campaign. The black dashed lines show the *Masters et al*.'s [2008] and *Kanani et al.*'s [2010] model bow shock and magnetopause positions for the typical solar wind dynamic pressure of 0.03 nPa as employed in section 2.1, from which it can be seen that the spacecraft is expected to have been located in the solar wind just upstream from the bow shock for an interval spanning apoapsis in both cases. The solid line segments on each trajectory indicate a 6 day interval in each case for which Cassini data are shown below in Figures 2 and 3, generally containing both magnetosheath and solar wind intervals as may be expected, but where HST obtained auroral images, while Cassini was continuously located in the solar wind over intervals of at least several hours. The position of the spacecraft at the light travel corrected center times of these ∼44 min HST imaging intervals are shown in Figure 1 by the open circles, three during the 2011 campaign on Revs 146/147 and four during the 2012 campaign on Rev 163.

Figure 2 shows relevant Cassini data for Revs 146/147, spanning days 90–95, inclusive, of 2011. Figure 2a shows a color-coded electron count rate spectrogram spanning energies between 0.6 eV and 28.75 keV, obtained by the electron spectrometer of the Cassini plasma spectrometer package [*Young et al.*, 2004], where we note that the intense component at lowest energies ∼1 eV is dominated by spacecraft photoelectrons. Figures 2b–2e show magnetic field data obtained from the fluxgate magnetometer [*Dougherty et al.*, 2004], where Figure 2b shows the field magnitude (nT) on a logarithmic scale, while Figures 2c–2e show the *x*, *y*, and *z* KSM field components, respectively. Figure 2f shows the clock angle of the field about the planet-Sun line, given by *θ* = arctan(*B*_*y*_/*B*_*z*_) defined between 0° and 180°. A clock angle in the range 0°–90° thus indicates a northward field potentially favorable for low-latitude reconnection and open flux production, while a clock angle in the range 90°–180° indicates a southward field unfavorable for low-latitude reconnection, but possibly favorable for lobe reconnection. Spacecraft KSM coordinates and radial distance (*R_S_*) are given at the day of year (DOY) boundaries at the bottom of the plot. Intermittent magnetosheath intervals are readily identified by the presence of warm electrons extending to several tens of electron volt energy with simultaneously elevated field strengths; these intervals having sharp boundaries as the spacecraft cross the bow shock. Otherwise, the spacecraft is located in the solar wind upstream from the shock.

The light travel corrected center times of the three ∼44 min HST imaging sequences, termed “visits,” that occurred in the interval shown in Figure 2 are marked by the vertical dashed lines, with visit identifiers H1–H3 being shown at the top of the plot (the excluded image mentioned above in section 1 is H4 occurring after the interval shown). It can be seen that Cassini had been located in the solar wind for a substantial prior interval, many hours, in each case. The red vertical bars overplotted in the magnetic field panels show the lagged 2.5 h intervals of IMF data relevant to the dayside auroral morphology, derived using equation 1 and the center time of each visit. In section 3, we employ slightly refined intervals corresponding to subsets of the image data obtained during each visit, but since these differ in center time by only ∼16 min in either direction from the overall center time employed here, this does not significantly affect the averaged IMF values derived. It can be seen that the IMF was directed near continuously northward in each of the intervals indicated by the red bands, thus potentially favorable for low-latitude reconnection and open flux production, with mean and standard deviation (SD) *B_z_* values of 0.16 ± 0.02, 0.13 ± 0.06, and 0.45 ± 0.27 nT, respectively. It can thus be seen that the largest *B_z_* field, by a factor of ∼3, occurred for visit H3. The corresponding averaged clock angles are 50° ± 6°, 52° ± 20°, and 67° ± 14°. These averaged IMF component values and clock angles are collected together in Table 1 for convenience and comparison, together with the computed lag times employed.

**Table 1:** Averaged Lagged IMF Components and Clock Angle for Each HST Visit

HST Visit	HST Visit Center Time h:min:s DOY/Year	Saturn Visit Center Time h:min:s DOY/Year	Lag Time/h^a^	IMF *B_x_*/nT^b^	IMF *B_y_*/nT^b^	IMF *B_z_*/nT^b^	IMF Clock Angle *θ*/deg^c^
H1	05:03:51	03:52:13	1.81	0.08 ± 0.02	0.19 ± 0.02	0.16 ± 0.02	50 ± 6
	91/2011	91/2011					
H2	03:23:19	02:11:41	1.68	−0.04 ± 0.06	0.17 ± 0.05	0.13 ± 0.06	52 ± 20
	93/2011	93/2011					
H3	06:30:29	05:18:51	1.53	−0.30 ± 0.14	−1.00 ± 0.10	0.45 ± 0.27	67 ± 14
	95/2011	95/2011					
I5	10:47:15	09:34:29	2.21	−0.15 ± 0.09	−0.07 ± 0.10	0.15 ± 0.08	33 ± 26
	92/2012	92/2012					
I6	10:48:56	09:36:12	2.21	−0.00 ± 0.03	−0.33 ± 0.05	−0.54 ± 0.03	148 ± 5
	93/2012	93/2012					
I7	10:45:49	09:33:07	2.19	−0.07 ± 0.05	−0.42 ± 0.04	−0.41 ± 0.06	134 ± 7
	94/2012	94/2012					
I8	13:54:13	12:41:33	2.14	−0.34 ± 0.03	0.26 ± 0.06	−0.18 ± 0.06	124 ± 14
	95/2012	95/2012					

a Uncertainties in lag time values are ∼±0.4 h, see section 2.1.

b Values given are the mean and standard deviation over 2.5 h lagged intervals based on the visit center time, see section 2.1.

c Values given are the mean and standard deviation obtained using the “directional statistics” approach of *Mardia and Jupp* [2000], appropriate to circular measure, determined over the same 2.5 h lagged intervals as the field values.

Figure 3 then shows Cassini data for Rev 163, spanning days 91–96, inclusive, of 2012, in the same format as Figure 2. The spacecraft is located in the magnetosheath at the start of the interval, unsurprisingly given the trajectory of the spacecraft shown in Figure 1, but passes across the bow shock into the solar wind early on day 92, with intermittent magnetosheath intervals occurring thereafter presumably during transient decreases in solar wind dynamic pressure. The light travel corrected center times of four HST visits are again shown by the vertical dashed lines marked with identifiers I5–I8 at the top of the plot, together with four lagged intervals relevant to the dayside auroras shown by the red bars, computed using equation 1. As in Figure 2, all these intervals were located exclusively in the solar wind, although the first of these, for visit I5, appears to have been disturbed by field fluctuations associated with the bow shock. For this case, the IMF was again near continuously northward, with mean and SD *B_z_* values of 0.15 ± 0.08 nT over the interval, similar to visits H1 and H2, and a highly variable clock angle of 33° ± 26° (Table 1). For the subsequent three visits, however, the IMF was directed consistently southward, unfavorable for low-latitude dayside reconnection, and had generally been so directed for a number of hours previously, although a brief interval of northward directed field had occurred shortly before the lagged interval for visit I8. The mean and SD *B_z_* values for I6–I8 are −0.54 ± 0.03, −0.41 ± 0.06, and −0.18 ± 0.06 nT (Table 1), such that the southward fields for I6 and I7 are of comparable strength to the strong northward field for H3, while the lesser southward field for I8 has a similar strength to the weaker northward fields of H1, H2, and I5. The corresponding clock angles for I6–I8 are 148° ± 5°, 134° ± 7°, and 124° ± 14°, respectively (Table 1).

## 3. Morphology and IMF Dependence of the 2011 and 2012 HST Campaign Auroras

### 3.1. HST Image Data and Display

The HST UV auroral images during the 2011 and 2012 Saturn campaigns were obtained using the Solar Blind Channel of the Advanced Camera for Surveys, the detector of which is a 1024 × 1024 pixel Multi-Anode Microchannel Array with highest throughput in the far-UV wave band 115–170 nm. The average resolution of the detector is ∼0.032 arcsec pixel^−1^, with an instrument point spread function (PSF) of 2 pixel. At Saturn's distance from Earth during these campaigns of ∼8.6 AU, this PSF translates to a spatial resolution in the images in the noon sector auroral region of ∼400 km east-west (∼0.1 h LT) and ∼1000 km north-south (∼1° latitude) due to the oblique view. We note that the latter value is of comparable order to the ∼1000–3000 km north-south spatial scales of the UVIS duskside arcs studied by *Radioti et al*. [2011, 2013], such that individual arc structures are at the limit of resolution in these HST images. Nevertheless, such emissions should certainly be observed by HST, forming a broad structured band of high-latitude UV aurora located in the noon to dusk sector.

During each campaign visit, 19 individual images were obtained over an interval of ∼44 min, each with an exposure time of 100 s. The first and last five images employed the F125LP filter that has a band pass of 125–170 nm, thus observing the Lyman and Werner band emissions of H_2_, but excluding H Lyman-*α* at 121.6 nm (to avoid image contamination by geocoronal emission when HST is not located in Earth's shadow). The 9 central images then usually employed the F115LP filter with a band pass of 115–170 nm, which thus includes H Lyman-*α* emission. All of these images show essentially similar features. However, here we choose to concentrate on the initial and final sets of 5 images using the F125LP filter, since these can then be compared with each other (but not directly with the central 9 images) to provide an indication of temporal variability and motion of auroral features, or the lack thereof, during a given visit. The data from each such set of 5 images have then been coadded to improve the signal to noise, such that here we examine two coadded images from each visit corresponding to the initial and final 5 image sets, each spanning an interval of ∼11 min, with ∼33 min between the two center times (i.e., ∼±16 min about the overall visit center time). For the ease of reference, the coadded image from the initial interval of each visit will be termed “image A” for that visit, while that from the final interval will be termed “image B.” We note that in the ∼11 min interval contributing to a single coadded image, a near rigidly corotating feature would rotate through only 0.4 h of LT, while in the ∼33 min between them, such a feature would rotate through a well resolved 1.2 h of LT.

**Figure 4:**
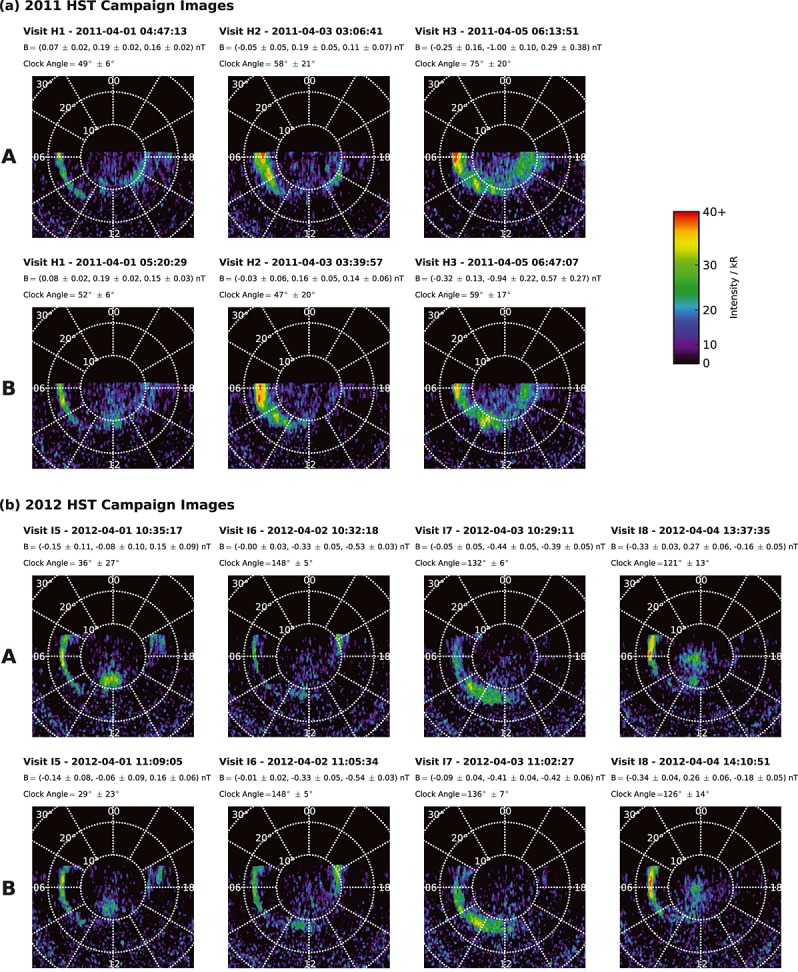
Set of projected HST images of Saturn's northern UV aurora from (a) the 2011 campaign (visits H1–H3) and (b) the 2012 campaign (visits I5–I8), during which Cassini was located in the solar wind upstream of Saturn's bow shock over intervals of at least several hours. The top rows of Figures 4a and 4b correspond to the five coadded F125LP filtered images at the start of each visit, while the bottom rows similarly correspond to the five coadded F125LP filtered images at the end of each visit. These are termed the “A” and “B” images, respectively, as marked in the figure. HST visit identifiers are given above each image, together with the date (year-month-day) and UT center time (h:min:s). Beneath this are shown the mean and SD values of the three KSM components of the IMF determined from the lagged 2.5 h intervals of Cassini data associated with the center time of each individual image, together with the corresponding mean and SD values of the IMF clock angle. The images are projected onto a polar grid assuming an auroral height of 1100 km above the 1 bar reference spheroid, the grid being shown by white dotted latitude circles at 10° intervals and white dotted longitude lines at 30° intervals (2 h LT), with noon shown at the bottom and dawn to the left. The images have been truncated somewhat past the dawn-dusk meridian to avoid pixels becoming overstretched on approaching the planetary limb. The same color-coded intensity scale, shown upper right, is employed for all images, saturated red at 40 kR.

The coadded images for the two campaigns are shown in Figures 4a and 4b, respectively, projected onto a latitude-longitude grid at a height of 1100 km above the 1 bar reference spheroid, the latter corresponding to the typical peak in the emission height profile [*Gérard et al*., 2009]. Noon is plotted at the bottom of each image, dawn to the left, and dusk to the right, with white dotted circles and radial lines showing 10° intervals of latitude and 30° intervals of longitude (2 h LT), respectively. Each image has been truncated past the dawn-dusk meridian to avoid overstretching the pixels as the HST view approaches the planetary limb, with a somewhat more expanded view being available for the 2012 campaign compared with 2011 due to the developing northern spring season at Saturn. The same color-coded intensity (kR) scale is used for all the images, shown upper right in the figure, saturated red at 40 kR. The coadded images obtained from the initial (A) and final (B) intervals of each visit of the 2011 campaign are shown in the top and bottom rows of Figure 4a, respectively, with the images from visits H1–H3 being shown in the columns from left to right. The images from visits I5–I8 of the 2012 campaign are shown in the same format in the top and bottom rows of Figure 4b. The header above each image gives the HST visit identifier, the date (year-month-day) and UT center time (h:min:s) of the image, together with the corresponding averaged lagged IMF components in KSM coordinates, and the averaged clock angle. These values have been determined over 2.5 h intervals lagged relative to the center time of the individual coadded images shown, but since, as indicated above, these center times are displaced by only ∼16 min either side of the overall center time marked in Figures 2 and 3, the values generally differ only marginally from those discussed in section 2.2 and shown in Table 1.

### 3.2. Case-by-Case Dayside Auroral Morphologies

In common with previous studies of Saturn's auroral morphology, Figure 4 shows that Saturn's dayside auroras usually consist of a relatively narrow arc spanning from the predawn sector toward noon, giving way to intermittent patchy forms at higher latitudes in the postnoon sector [e.g., *Gérard et al*., 2004, 2005; *Grodent et al*., 2005]. We now examine and discuss these emissions on a case-by-case basis before drawing overall conclusions in subsequent subsections.

In the images from visit H1 shown in Figure 4a, the dawn arc is centered near ∼17° colatitude, and is truncated prenoon near ∼10 h LT. At later LTs, the emissions then step poleward, forming a broader, weaker, spatially structured band spanning ∼10° colatitude that extends beyond the dusk meridian. The detailed form of the latter emissions is seen to change somewhat in the ∼33 min between images A and B, but the overall position appears to be fixed to a first approximation, with notable patches near to noon as well as in the predusk sector. Comparing this morphology with those presented, e.g., by *Radioti et al*. [2011, 2013] and *Badman et al*. [2012, 2013], it seems reasonable to suggest that the noon to dusk emissions correspond to the similarly located high-latitude structured auroras observed in the UVIS and VIMS data, which lie at the limit of resolution of individual “arc” features in these HST images as discussed in section 3.1. The association of these emissions with dayside reconnection-related phenomena is supported in the present case by the positive IMF *B_z_* known to have been prevailing over an extended prior interval, having a mean concurrent value of ∼0.16 nT with a mean clock angle of ∼50° (Table 1).

In the images from visit H1 shown in Figure 4a, the dawn arc is centered near ∼17° colatitude, and is truncated prenoon near ∼10 h LT. At later LTs, the emissions then step poleward, forming a broader, weaker, spatially structured band spanning ∼10° colatitude that extends beyond the dusk meridian. The detailed form of the latter emissions is seen to change somewhat in the ∼33 min between images A and B, but the overall position appears to be fixed to a first approximation, with notable patches near to noon as well as in the predusk sector. Comparing this morphology with those presented, e.g., by *Radioti et al*. [2011, 2013] and *Badman et al*. [2012, 2013], it seems reasonable to suggest that the noon to dusk emissions correspond to the similarly located high-latitude structured auroras observed in the UVIS and VIMS data, which lie at the limit of resolution of individual “arc” features in these HST images as discussed in section 3.1. The association of these emissions with dayside reconnection-related phenomena is supported in the present case by the positive IMF *B_z_* known to have been prevailing over an extended prior interval, having a mean concurrent value of ∼0.16 nT with a mean clock angle of ∼50° (Table 1).

The auroral morphology during visit H2, for which the mean concurrent IMF *B_z_* was slightly weaker at ∼0.13 nT with a clock angle of ∼52°, is similar to H1 (Table 1). The brighter wider dawn arc in this case, centered near ∼14° colatitude, is again truncated near ∼10 LT, stepping poleward to weaker variable patchy emissions at around ∼10° colatitude near noon (particularly image B) and at later LTs extending toward dusk (particularly image A). The noon to dusk emissions are somewhat less extensive than those observed during visit H1, however possibly resulting from the weaker IMF *B_z_* prevailing and the lesser time for which the IMF had been northward pointing prior to the visit (Figure 2).

Visit H3 then occurred in association with the largest positive IMF *B_z_* in this data set by a factor of ∼3, ∼0.45 nT with a clock angle of ∼67° (Table 1), for which it can be seen that auroras are continuously present across the full range of LTs in both images. Structured dawn arc emissions centered at ∼14° colatitude near dawn move poleward to ∼12° in the prenoon sector and then give way postnoon to a broad band of high-latitude emissions poleward of ∼10° that extend past the dusk meridian. Similar features are observed in both H3 images, although in image B, the emission near dusk appears to have contracted slightly poleward compared with image A. Again, the broad band of postnoon emissions may well correspond to a multiple bifurcated oval, such as those shown in Figures 1 and 2 of *Radioti et al*. [2011, 2013], the detailed structure of which remains unresolved here.

Continuing with the images from the 2012 HST campaign shown in Figure 4b, visit I5 also occurred during an interval of northward directed IMF, with a mean lagged *B_z_* of ∼0.15 nT and a clock angle of ∼33°, this IMF *B_z_* value being similar to the weaker northward field cases H1 and H2 (Table 1). In this case a narrow dawn arc centered at ∼16° colatitude extends continuously from beyond the dawn meridian to ∼9–10 LT, giving way at later LTs to a high-latitude patch of emission poleward of ∼10° in the noon sector. In image A, the patch is bright and extends in a ∼3 h LT band centered near noon, while in image B, it is less intense with a center moved toward the prenoon sector. No significant emission is present at later local times in the predusk sector. However, structured forms are observed in both images A and B at lower latitudes, ∼13° colatitude, in the postdusk sector, a region that was inaccessible to HST during the 2011 campaign. This morphology appears similar to that for visit H1 in Figure 4a occurring under similar northward IMF *B_z_* conditions, in which the dawn arc is also truncated near ∼10 LT, giving way at later LTs to a similar patch of high-latitude auroras near to noon which is somewhat separated from the predusk emission, particularly in image B.

Unlike the four cases discussed above, the three remaining images from the 2012 campaign all occurred under continuously southward IMF conditions as outlined in section 2.2, with stronger concurrent negative IMF *B_z_* values during visits I6 and I7 and a weaker negative *B_z_* during I8. For visit I6, for which the mean lagged *B_z_* value and clock angle were ∼ −0.54 nT and ∼148° (Table 1), respectively, it can be seen that a narrow dawn arc is centered at ∼17° colatitude near the dawn meridian. The emission then dims and becomes more scattered in the midmorning sector between ∼8 and ∼10 h LT, before brightening again and moving poleward to ∼12° near noon, finally terminating postnoon near ∼13 h. The auroral enhancement near to noon has some similarity to that observed for northward IMF in visits H1, H2, and I5, but in this case it is centered at lower latitudes and appears to be joined by weaker structured emission to the arc spanning dawn, particularly in image B. We thus infer that the noon emission in this case forms a continuation of the dawn arc into the noon sector, an interpretation also supported by examination of the emissions for visit I7 described below, observed under similar IMF conditions. We note that significant spatial structuring is typical of dawn arc emissions in the midmorning hours [*Meredith et al*., 2013]. There are then no significant emissions for visit I6 in the post noon to dusk sector, although emissions are again present past the dusk meridian, similar to those observed (for northward IMF) in visit I5.

The IMF conditions for visit I7 were similar to those for I6, as just noted, with a mean lagged *B_z_* value and clock angle of ∼ −0.41 nT and ∼134° (Table 1), respectively. In this case a wider brighter band of variable dawn arc emission stretches continuously from predawn and terminates postnoon at ∼13 h LT. The emission is centered near ∼15° colatitude at dawn and moves poleward to ∼12° near noon. The LT extent and latitudinal variation of this emission thus has much in common with that observed for visit I6, but is now clearly continuous in LT from predawn to postnoon, thus supporting the view that the dawn-to-noon emissions for I6 also represent extended but variable dawn arc emissions under similar IMF conditions, as indicated above. Also as for visit I6, the postnoon region is then devoid of significant emissions and remains, so in this case beyond the dusk meridian throughout the field of view.

For visit I8, with a weaker mean southward field of ∼ −0.18 nT and a clock angle of ∼124° (Table 1), we again observe a narrow bright dawn arc centered near ∼15° colatitude near dawn, which appears to extend into weaker structured forms in the midmorning sector that vary somewhat between images A and B and approach ∼10° colatitude prenoon. As for the other visits with negative IMF *B_z_*, there are again no patchy emissions present between noon and dusk extending around the high-latitude oval. However, a well-defined patch of polar emission now lies poleward of the structured dawn arc, near stationary in images A and B, principally on the dawn side of the noon-midnight meridian, extending to the magnetic/spin pole itself. We suggest that this patch is likely located on open field lines and is associated with high-latitude “lobe” reconnection between the southward directed magnetosheath field and open field lines in the northern tail lobe, as previously discussed for Saturn by *Bunce et al.* [2005] and *Gérard et al*. [2005]. This suggestion will be discussed further in relation to the noon sector auroral morphologies for positive IMF *B_z_* in section 3.5.

### 3.3. Dawn Arc

In the following subsections, we now compare the auroral features observed during the seven HST imaging visits, focusing on possible IMF dependencies, and begin by considering the dawn arc. Figure 4 shows that the dawn arc is a persistent feature in this data set, being present in all of the images examined, although we note that it is very occasionally absent in the wider Saturn auroral archive. It can also be seen however that it exhibits somewhat variable properties from visit to visit. While the arc is typically centered near ∼15° colatitude in the dawn sector, its position at a given LT clearly varies by a few degrees over the 1–2 day intervals from visit to visit, as noted in section 3.2 above, though not discernibly over the ∼33 min intervals between the two images from a given visit. In addition, the latitudinal thickness of the arc is somewhat variable over the range ∼1° to ∼4°, while the intensity varies from small values up to ∼40 kR. As mentioned in section 3.2, it also often exhibits spatially structured subcorotating patches of emission in the midmorning hours, previously analyzed by *Meredith et al*. [2013] using the 2009 equinoctial campaign images, that are particularly evident here in the image pairs for visits H2, H3, and I7.

Examination shows however that none of these features are simply related to the mean lagged concurrent IMF vectors determined here, for any KSM component. For example, if we consider the subset of images with positive IMF *B_z_*, H1–H3 and I5, these contain examples of slightly expanded (H1) and contracted (H3) latitudinal positions, as well as wide and bright (H2) and narrow and dimmer (I5) arcs. The subset with negative IMF *B_z_*, I6–I8 shows similar variability, such that these features clearly do not depend on the concurrent IMF *B_z_*. The same conclusion can be drawn by considering the image subsets, e.g., with positive (H1, H2, and I8) and negative (H3 and I5–I7) concurrent IMF *B_y_* (Table 1). One possible exception however concerns the LT extent of the dawn arc in the near-noon sector, which is often truncated prenoon near ∼10 h LT for northward IMF (visits H1, H2, and I5), but can extend postnoon to ∼13 h LT for southward IMF (visits I6 and I7).

Overall, however, it thus appears that the main properties of the “quiet time” dawn arc do not depend on the value of the concurrent IMF. It is known on a statistical basis that the dawn emissions are modulated by the “planetary period oscillations” observed ubiquitously in Saturn's magnetosphere [*Nichols et al*., 2010]. However, examination shows that the variations found in the individual examples considered here are not clearly related to the northern oscillation phase either. The origin of these dawn arc variations thus requires further study.

### 3.4. Noon to Dusk Emission

While the dawn arc is thus a persistent, if somewhat, variable feature in these images, the discussion in section 3.2 shows that the emissions in the noon to dusk sector are considerably more intermittent. The images from visits H1–H3 and I5, associated with northward IMF, all show the presence of high-latitude emissions in this sector, beyond the LT extent of the dawn arc. However, the form of these emissions varies significantly from visit to visit, and somewhat over the ∼33 min intervals between images A and B of a given visit, thus indicating the presence of a dynamic phenomenon varying on time scales down to a few tens of minutes. In addition, the most extensive of these noon to dusk emissions has been found to be associated with the strongest positive IMF *B_z_*. However, such emissions are conspicuously absent for visits I6–I8, i.e., for all three cases for negative IMF *B_z_*.

Based on this IMF *B_z_* dependency, and on the variable nature of these emissions observed both within visit and from visit to visit, it seems reasonable to suggest that these emissions are associated dayside reconnection, related to the structured flows, field-aligned coupling currents, and precipitation associated with dayside events occurring at various locations on the magnetopause, observed at various stages of their few hour auroral development. Indeed, it seems likely that these emissions are manifestations of the same high-latitude noon to dusk auroral phenomenon reported by *Radioti et al*. [2011, 2013] and *Badman et al*. [2013], though observed here with lesser spatial resolution. These authors suggested that the structured auroras observed in their images are associated with dayside reconnection, a proposal that is strongly supported here by the observed IMF *B_z_* dependency.

### 3.5. Polar Emission

In the discussion of individual visits in section 3.2, we suggested that the polar patch of emission observed on visit I8 is associated with lobe reconnection occurring under the southward IMF conditions then prevailing. We note however a superficial similarity with the auroral morphology observed for visit I5, and also for H1, in which a well-defined patch of emission also appears at relatively high latitudes, ∼10° colatitude and poleward thereof, centered near to noon. Without additional information, it might seem reasonable to suggest that these patches too could be associated with lobe reconnection. However, the concurrent northward direction of the IMF, with a clock angle of only ∼33° for visit I5, the most northward of all the IMFs in this data set (Table 1), shows that this cannot be the case. Rather, as in section 3.2, these emissions are suggested to form part of the continuum of variable high-latitude patchy auroras that form the noon to dusk oval for northward IMF, related to similarly variable lower latitude reconnection events occurring at the magnetopause. We note that for visits H1 and I5 for northward IMF, the dawn arc is truncated in the prenoon sector before the emission steps poleward near noon and at later LTs. This suggests an auroral oval that is located mainly on closed field lines in the dawn sector (noting the lack of IMF dependence of the dawn arc) but moves poleward onto newly opened field lines in the noon to dusk sector associated with lower latitude reconnection. For visit I8 for southward IMF, however, the polar patch of emission lies near stationary, principally in the dawn-to-noon sector poleward of the midmorning extension of the dawn arc, reaching to much higher latitudes than for the northward IMF cases, encompassing the magnetic/spin pole itself. These results therefore show that care must be taken when considering the physical origins of dayside emissions at Saturn.

## 4. Summary and Discussion

In this paper we have examined a unique set of HST observations of Saturn's dayside auroras during which the Cassini spacecraft was located in the solar wind just upstream from Saturn's bow shock, measuring the IMF over concurrent intervals of at least several hours. Confining the study to intervals exhibiting usual “oval” auroral morphologies, as opposed to rarer disturbed conditions when the dawn auroras expand poleward to high latitudes, we have found seven such simultaneous intervals, all of which occurred during the postequinox 2011 and 2012 HST auroral campaigns, thus involving the northern UV auroras.

As in previous studies, the dayside auroras generally exhibit a relatively narrow arc extending continuously from predawn toward noon, together with intermittent broader patchy forms at higher latitudes between noon and dusk. In the images examined here, the dawn arc is a persistent feature, but centered variably in the range ∼12°–17° colatitude, with variable latitudinal width and intensity. None of these variations has been found to be related to suitably lagged and averaged KSM components of the concurrent IMF (nor indeed to the concurrent phase of the northern planetary period oscillations). Future study of the origins of this variability is thus warranted. However, auroral features at LTs beyond the dawn arc in the noon to dusk sector show a consistent dependency on IMF *B_z_*. Specifically, all four cases with positive IMF *B_z_* show the presence of patchy auroras in the noon to dusk sector, more broadly distributed in latitude than the dawn arc and located at higher latitudes, ∼10° colatitude and poleward thereof, while none of the three cases with negative IMF *B_z_* show similar features. The three cases with weaker positive IMF *B_z_* exhibit variable patchy high-latitude forms a noon patch and/or a patchy arc in the predusk sector, while the case with a significantly stronger IMF *B_z_*, by a factor of ∼3, shows continuous emissions from noon to beyond the dusk meridian. All these cases also show some variation in the postnoon emissions over the ∼33 min interval between the initial and final images obtained on a given HST visit, thus indicating evolution of the related physical process on such time scales. While the images for negative IMF *B_z_* show no such auroras, one (for visit I8) does show the presence of a prominent polar patch centered in the dawn-to-noon sector poleward of a structured dawn arc, extending to the magnetic/spin pole itself.

The comparison with the auroral morphologies discussed using UVIS data by *Radioti et al.* [2011, 2013] and *Badman et al.* [2013] suggests that the patchy high-latitude auroras observed between noon and dusk correspond to the structured forms in this sector that these authors propose to be associated with dayside reconnection, though here observed with lesser spatial resolution. The exclusive association found here of such emissions with northward IMF, favorable for low-latitude dayside reconnection and open flux production at Saturn, provides support for this view. In this scenario, the variability of the forms observed under this condition, both during a visit and from visit-to-visit, can be ascribed to observations of reconnection-related phenomena occurring at differing locations on the magnetopause, and in varying stages of their few hour evolution from the dayside magnetopause into the tail. The confinement of such features to the noon to dusk sector is likely due to the suppression of magnetopause reconnection at dawn by the strong flow shear across the boundary at such LTs, an effect that is reduced in the postnoon sector [*Desroche et al.*, 2013]. We emphasize that if the reconnection events inferred from the auroral observations by *Radioti et al.* [2011, 2013] and *Badman et al.* [2013] have similar character to FTEs at Earth, as seems likely, the time scales of these events will be few hours and not few minutes, as a result principally of the much larger spatial scale of the Saturn system. The failure of *Lai et al*. [2012] to detect few-minute Earth-like FTEs at Saturn is then not a major surprise. Along related lines, the lack of correspondence noted in section 3 between the latitude of the auroras, either the dawn arc or the postnoon emissions, and the concurrent few hour mean value of IMF *B_z_* is also not surprising, since the time scale for open flux accumulation in the system that influences the size of the open field region and hence that of the auroral oval is usually days and not hours [*Jackman et al*., 2004; *Badman et al*., 2005]. Modest, few degree, changes in latitude are observed in this data set on the 1–2 day intervals between visits, but are not discernible on the ∼30 min intervals during visits.

The polar patch observed in one case (visit I8) for relatively weak southward IMF is then suggested to be associated with intermittent lobe reconnection on open field lines, following the earlier discussions of *Bunce et al.* [2005] and *Gérard et al*. [2005], and the additional possible examples shown by *Badman et al.* [2012, 2013]. The prenoon preference of this patch in the present case may be associated with the positive IMF *B_y_* conditions prevailing (Table 1), which via the antiparallel field condition would favor the lobe reconnection prenoon in the northern hemisphere and postnoon in the southern hemisphere at Saturn, similar to but opposite in sense to the effect observed at Earth due to the opposite polarity of the planetary dipole field [e.g., *Milan et al*., 2000b]. We would not expect plasma rotation effects to play such a significant role in the lobe reconnection process at polar latitudes as they appear to do in open flux production nearer the equator.

We conclude by noting that the above discussion of the physical origins of these emissions is in excellent accord with the results of a parallel study of these images by E. S. Belenkaya et al. (submitted manuscript, 2014). In order to examine the auroral source regions, these authors employed the “paraboloid” model of Saturn's magnetosphere to magnetically map the regions of bright auroral emission from the northern ionosphere into the magnetosphere. The magnetic model includes the internally generated field of the planet, typical ring and tail current fields, the shielding effect of the current flowing on a parabolic magnetopause, and a partially penetrating IMF derived from the concurrent Cassini field data essentially as in section 2.1. They found that the dawn arcs map typically from near the inner edge of the dawn-sector ring current at ∼7 *R_S_* radial distance, outwards to either the center of the ring current at ∼10 *R_S_* for narrow arcs, or to its outer edge near ∼15 *R_S_* for the broader dawn emissions, or to the outer magnetosphere close to the magnetopause for the broadest dawn emissions such as those that occur for visit H3. These auroras thus lie essentially wholly on closed field lines in the outer part of the dawn magnetosphere and are thus likely to be associated with flow shears and pressure gradients within the hot ring current plasma injected from the nightside region. The lack of dependence on the “concurrent” IMF found here is thus unsurprising. The reason for the strong dawn-dusk asymmetry in these emissions, with no equivalent emission being observed in the postnoon sector for either northward or southward IMF, at least within the sensitivity of the present HST image data, is unclear. However, it could possibly relate to the persistent dawn-dusk asymmetry associated with the Vasyliunas flow cycle, with slow outflows of mass-loaded flux tubes down the tail at dusk, and rapid returns of pinched-off flux tubes via dawn [*Vasyliunas*, 1983; *Cowley et al*., 2004a, 2004b].

The higher-latitude patchy forms observed for northward IMF in visits H1–H3 and I5 in the noon to dusk sector however are all found to straddle the model open-closed field line boundary, consistent with our discussion of processes connected with lower latitude reconnection and open flux production at the dayside magnetopause. We also note that the near-noon emissions for southward IMF, which can sometimes adopt a patchy form, particularly for visits I6 and I8, were found to be located principally on closed model field lines near the outer boundary of the ring current and in the outer magnetosphere. This supports our conclusion in section 3.2 that these emissions are associated with the dawn arc and its termination in the noon (I8) to postnoon (I6 and I7) sector under these conditions. The postdusk arcs seen for northward and southward IMF during visits I5 and I6 are also located principally on closed model field lines. Finally, the polar emissions observed on visit I8 are found to map wholly to open field lines in the model, consistent with our suggested “lobe reconnection” origin. The results of E. S. Belenkaya et al. (submitted manuscript, 2014) are thus found to be fully consistent with the discussion given here.

Overall, the HST auroral images examined here provide new evidence of significant IMF dependence of Saturn's dayside auroras, though using only the limited data set of seven simultaneous observation intervals with usual auroral morphologies presently available. Clearly, this study should be followed by further investigation if more such data sets became available in future.
